# Metal Organic Frameworks Based Materials for Heterogeneous Photocatalysis

**DOI:** 10.3390/molecules23112947

**Published:** 2018-11-12

**Authors:** Shu-Na Zhao, Guangbo Wang, Dirk Poelman, Pascal Van Der Voort

**Affiliations:** 1 Department of Chemistry, Center for Ordered Materials, Organometallics and Catalysis (COMOC), Ghent University, Krijgslaan 281 (S3), 9000 Gent, Belgium; shuna.zhao@Ugent.be (S.‐N.Z.); Guangbo.Wang@UGent.be (G.W.); 2 LumiLab, Department of Solid State Sciences, Ghent University, Krijgslaan 281 (S1), 9000 Gent, Belgium; Dirk.Poelman@UGent.be

**Keywords:** metal-organic framework, heterogeneous photocatalysis, solar energy

## Abstract

The increase in environmental pollution due to the excessive use of fossil fuels has prompted the development of alternative and sustainable energy sources. As an abundant and sustainable energy, solar energy represents the most attractive and promising clean energy source for replacing fossil fuels. Metal organic frameworks (MOFs) are easily constructed and can be tailored towards favorable photocatalytic properties in pollution degradation, organic transformations, CO_2_ reduction and water splitting. In this review, we first summarize the different roles of MOF materials in the photoredox chemical systems. Then, the typical applications of MOF materials in heterogeneous photocatalysis are discussed in detail. Finally, the challenges and opportunities in this promising field are evaluated.

## 1. Introduction

A significant and ongoing challenge is the increasing pollution associated with the highly increased global energy consumption [1,2]. The exploration of more sustainable and clean energy sources has become an extremely important and challenging task that humanity needs to address urgently, as CO_2_ and other pollutants have a detrimental effect on our climate and health. As an abundant and sustainable energy source, solar energy represents the most attractive clean energy source to replace fossil fuels. Photosynthesis is a transformation process where a plant can harvest solar radiation and convert carbon dioxide (CO_2_) and water (H_2_O) into carbohydrates. Great efforts have been devoted to developing artificial photosynthetic systems (or simply photocatalytic systems) for chemical transformations by using inorganic or/and organic materials. The first photocatalytic system was achieved by Fujishima and Honda in their pioneering work on water splitting with TiO_2_ under UV light irradiation [3]. Since then, various types of materials have been studied and employed for the application of photocatalysis [4]. However, the nanostructures and functionalization of these materials need to be optimized to maximize utilization of sunlight as well as increase their photocatalytic performance.

The classical photocatalytic process consists of the following three fundamental steps: (1) photosensitizers absorb solar irradiation to create charge-separated excited states; (2) redox equivalents (mobile electrons and holes) are produced and migrate to catalytic centers; (3) redox equivalents react with substrates at the reactive centers. Therefore, an excellent photocatalyst ought to possess the following features: (1) strong absorption of sunlight; (2) a long lifetime of excited state; (3) high yield of charge-separated states; and (4) good charge mobility. 

Metal organic frameworks (MOFs) are a new class of functional hybrid crystalline materials, which are assembled by metal centers or clusters and organic ligands, forming one-, two-, or three-dimensional extended coordination networks. The structural diversity, high porosity, framework flexibility, large surface area, as well as tunable pore surface properties provide them with unique functions for diverse applications. This includes luminescence [5,6,7], gas separation and adsorption [8,9,10], magnetism [11], chemical sensing [12,13,14], proton conductivity [15,16], energy storage and conversion [17,18,19], and biomedicine [20,21]. In addition, heterogeneous catalysis is one of the most distinct fields of MOFs because of their uniform, tailorable, controllable and post-modifiable porous structures [22,23,24]. In recent years, MOFs have also emerged as promising candidates for photocatalysis. Firstly, MOFs can integrate photosensitizers and catalytic components in a single material by immobilizing the active sites on metal nodes, organic linkers, or encapsulated guest molecules inside the pores. The limitless choices of metal nodes and organic linkers in MOFs offer the possibility to improve the use of the visible spectrum of sunlight. Secondly, the high porosity of MOFs allows fast transport and diffusion of substrates and products from catalytic sites. The well-defined crystalline nature of MOFs provides a unique platform to investigate the energy transfer mechanism of the photocatalytic process, which is difficult to study in other photocatalytic systems. Thirdly, unlike homogeneous photocatalysts, MOFs can be easily separated from the reaction systems and can be reused multiple times. Therefore, it will extend the lifetime of the photocatalysts and reduce waste and contamination.

The thermal and chemical stabilities of MOFs is an important part of why they can be used as catalysts or catalyst hosts. MOF-based materials should be stable under catalytic conditions, particularly in water, and be resistant to moderately acidic or basic solutions. In recent years, a series of robust MOFs have been reported. For example, Zr-based MOFs showed high stability in water due to the strong coordination between the Zr nodes and the organic linkers [25]. Zeolitic imidazolate frameworks (ZIFs), which are constructed by the use of anionic, nitrogen-containing ligands, are stable in water [26]. This can be ascribed not only to the strong bond of the nitrogen-containing linkers with metal nodes, but also to the effective physical shielding of metal nodes by the coordinated nitrogen-containing linkers. In addition, decorating fluorinated, sulfonic, or phosphonate substituents in the organic linkers can contribute to stability in water [27].

In this review, we first discuss the different roles that MOFs play in photocatalytic systems, such as photocatalysts, hosts, or precursors. Then, we summarize and highlight the latest developments of MOF materials in photocatalytic applications, including degradation of pollutants, organic transformations, CO_2_ reduction and water splitting. This review presents a comprehensive discussion and investigation of the rational design of MOF-based photocatalysts to provide insights for the future developments of novel and highly efficient photocatalysts.

## 2. The Functions of MOFs in Photocatalytic Systems

### 2.1. MOFs as Photocatalysts

**Figure 1:**
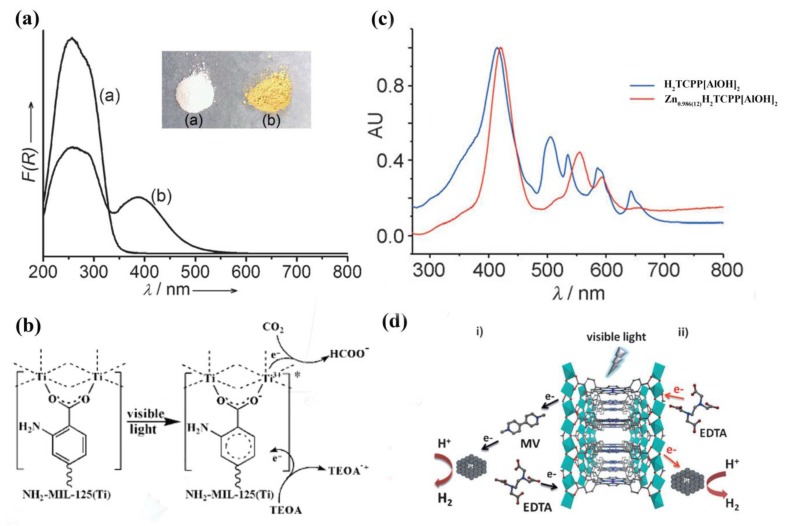
(**a**) UV/Vis spectra of (a) MIL-125(Ti) and (b) NH2-MIL-125(Ti). The inset shows the samples. (**b**) Proposed mechanism for the photocatalytic CO_2_ reduction over NH2-MIL-125(Ti) under visible light irradiation. Reproduced with permission from Reference [33]. Copyright 2012 Wiley-VCH Verlag GmbH & Co. KGaA, Weinheim (**c**) UV/Vis solid-state absorption spectra of H_2_TCPP[AlOH]_2_ and Zn_0.986(12)_H_2_TCPP[AlOH]_2_. (**d**) The photocatalytic reaction using Zn_0.986(12)_H_2_TCPP[AlOH]_2_. (i) Reaction involving Zn_0.986(12)_H_2_TCPP[AlOH]_2_, methyl viologen, colloidal platinum, and sacrificial EDTA. (ii) Reaction involving Zn_0.986(12)_H_2_TCPP[AlOH]_2_, colloidal platinum, and sacrificial EDTA. Reproduced with permission from Reference [36]. Copyright 2012 Wiley-VCH Verlag GmbH & Co. KGaA, Weinheim.

Previous work using MOFs for photocatalysis was mainly based on their semiconducting properties because MOFs are a class of analogues of inorganic semiconductors [28,29]. In 2007, Garcia’s group provided experimental evidence for the behavior of MOF-5 as a semiconductor [30]. The Zn_4_O clusters of MOF-5 can be considered as semiconductor dots, which are isolated and distributed regularly in the framework. The terephthalate linkers can absorb light to bring these dots to their excited state, and then transfer the photoinduced electrons to Zn^2+^ through ligand-to-metal charge transfer (LMCT). A charge-separated state of MOF-5 was observed and the band gap was estimated to be 3.4 eV. Since then, a variety of MOFs have been employed for photocatalysis, such as UiO-66(Zr) [31] and MIL-125(Ti) [32]. These MOFs are typically used for the degradation of organic pollutants. They showed low efficiency of light energy utilization due to the large effective band gap. This can be improved by modification of the organic linkers. Li and coworkers synthesized an amine-functionalized MIL-125(Ti) homologue Ti_8_O_8_(OH)_4_(BDC-NH_2_)_6_ (NH_2_-MIL-125(Ti)) (BDC-NH_2_ = 2-amino-benzene-1,4-dicarboxylate) simply by using BDC-NH_2_ as the organic linker [33]. The light energy absorption of NH_2_-MIL-125(Ti) is significantly changed by the amino functionality (Figure 1a). NH_2_-MIL-125(Ti) exhibits an absorption band edge at around 550 nm, falling in the visible region, while MIL-125(Ti) shows an absorption edge at 350 nm. The significant red-shift in light absorption enhances the photocatalytic activity for visible light irradiation. The photocatalytic reduction of CO_2_ is then realized by using NH_2_-MIL-125(Ti) as the photocatalyst under visible light irradiation (Figure 1b). A similar strategy was also used for synthesizing NH_2_-UiO-66 and NH_2_-MIL-101(Cr), which were used as photocatalysts for hydrogen production from water [34,35]. Hereafter, much research was dedicated to synthesizing refined organic linkers with a better photon antenna effect to improve the photocatalytic activity of MOF materials. 

Porphyrins are known to be light harvesting compounds and have been used as building blocks to synthesize MOFs with an excellent photocatalytic performance [37]. Rosseinsky and coworkers reported a red porphyrin-based MOF H_2_TCPP[AlOH]_2_(DMF_3_-(H_2_O)_2_) by using the free-base *meso*-tetra(4-carboxyl-phenyl) porphyrin (H_2_TCPP) as the organic linker [36]. Due to the zero occupancy of the Al atom at the center of porphyrin ligand, H_2_TCPP[AlOH]_2_ reacted with anhydrous Zn(AC)_2_ through porphyrin metalation, generating a purple material Zn_0.986(12)_H_2_TCPP[AlOH]_2_. H_2_TCPP[AlOH]_2_ exhibits a strong absorption band at 415 nm, belonging to the S_0_→S_2_ absorption process. Another four Q bands at lower energies were originated from the π-π* transitions in the free-base porphyrin ligand (Figure 1c). After porphyrin metalation, Zn_0.986(12)_H_2_TCPP[AlOH]_2_ exhibits a slight red shift in the absorption edge at 425 nm. Due to the higher symmetry of the now metalated material, there are only two Q bands left. As the porphyrin-based MOFs are photocatalytically active in the visible light region, the authors evaluated the photocatalytic performance of the two MOFs for hydrogen evolution from water (Figure 1d). 

Another class of famous building blocks for MOFs is dye molecules, especially metallo-organic dyes such as Ru(bpy)_3_^2+^, [Ir(ppy)_2_(bpy)]^+^ (bpy = 2′2-bipyridine, ppy = 2-phenylpyridine) [38,39]. Due to their strong visible light absorption and long-lived excited states, they have been used as homogeneous photocatalysts. The incorporation of the photoredox-capable dyes into MOF frameworks could broaden and deepen the photocatalytic applications of MOFs. Meanwhile, the self-quenching induced by aggregation of dye-photocatalysts in homogeneous systems can be avoided thanks to the highly ordered distribution of dye-photocatalysts in MOF structures. 

### 2.2. MOFs as Co-Catalysts and/or Hosts

MOFs can act as hosts for photoredox species. They benefit from their high porosity, which provides additional possibilities for photocatalytic applications. Photocatalytically active species can be encapsulated into the pores of MOFs as guest molecules, provided that they obtain the right properties. These host MOFs display enhanced photocatalytic performance compared to homogeneous photocatalysts. This effect is due to the isolation of guest molecules and the mutual effect on the framework of MOFs. MOFs can act either as mere hosts or participate in photocatalytic processes. The high porosity of MOFs provides the necessary space for the interaction between the embedded catalytically active species and the substrates. Moreover, the very uniform pore size can result in reactant or product shape selectivity. Among the species that can be encapsulated are precious metals, semiconductor nanoparticles (NPs), as well as molecule catalysts which obtain the appropriate size [40,41]. 

**Figure 2:**
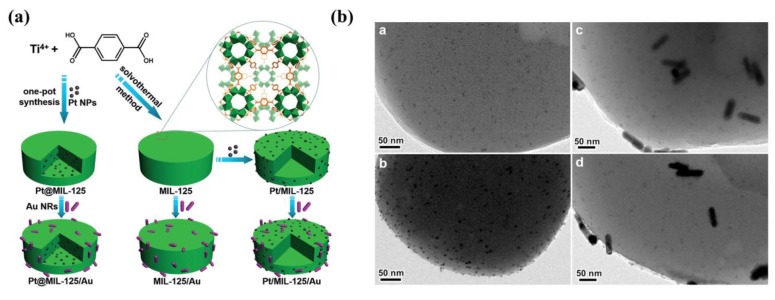
(**a**) Schematic illustration showing the synthesis of Pt@MIL-125/Au and the corresponding Pt/MIL-125/Au and MIL-125/Au analogues. (**b**) Typical TEM images of (a) Pt@MIL-125, (b) Pt/MIL-125, (c) Pt@MIL-125/Au, and (d) Pt/MIL-125/Au. Reproduced with permission from Reference [46]. Copyright 2017 Wiley-VCH Verlag GmbH & Co. KGaA, Weinheim.

The encapsulation of precious metals (Such as Pt, Pd and Au) into MOFs can inhibit the recombination between the photogenerated electrons and holes [42,43]. Due to the formation of a Schottky barrier at the junction between MOFs and precious metals, the photogenerated electrons in the conduction band (CB) of MOFs can transfer to the precious metals. This results in the efficient separation of the photogenerated charge carriers [44]. This way, the photocatalytic performance of MOF-based materials can be significantly enhanced. In 2014, Li and coworkers reported a series of M/NH_2_-MIL-125(Ti) materials (M = Pt and Au), which were used for CO_2_ reduction under visible light irradiation [45]. The Pt/NH_2_-MIL-125(Ti) exhibits an improved photocatalytic performance compared to NH_2_-MIL-125(Ti), while Au exhibits a detrimental effect on this reaction. The hydrogen-assisted formed Ti^3+^ plays a positive role in photocatalytic formate production. However, the ESR signal of Ti^3+^ was only observed in Pt/NH_2_-MIL-125(Ti). Neither in Au/NH_2_-MIL-125(Ti) nor in pure NH_2_-MIL-125(Ti) was the ESR signal observed, resulting in different effects on CO_2_ reduction. Interestingly, Jiang and coworkers incorporated uniform Pt NPs into MIL-125(Ti), followed by coating with Au nanorods (NRs) on the MIL-125(Ti) surface to form Pt@MIL-125(Ti)/Au (Figure 2) [46]. This integrated both the plasmonic effect of Au nanorods and a Schottky junction in a single MOF for the first time. The spatial separation of Au NRs and Pt NPs by MIL-125(Ti) steers the charge flow and greatly accelerates the charge migration, resulting in an exceptionally high photocatalytic performance of H_2_ evolution under visible light irradiation. These results show that with the appropriate use and distribution of precious metals in MOF materials, this is a very promising approach to improve the photocatalytic performance of MOFs.

Semiconductor NPs such as TiO_2_, CdS and ZnO, have a strong quantum-size effect and exhibit high photocatalytic activity [47]. Semiconductor NPs also have several disadvantages, including the aggregation in reactions, high recombination rate of photogenerated electron-hole pairs, and difficult separation from reaction systems. These are limiting their possible photocatalytic applications when used individually [48]. Hybrid materials between semiconductor NPs and MOFs do not only possess advantages from both two components, but also can overcome their individual limitations. The pioneering research on semiconductor NPs-MOF hybrid materials including CdSe/ZnS-MOF [49] and GdN/ZIF-8 [50], has mainly focused on enhancing light harvesting. Recently, Zhu and coworkers solvothermally synthesized a novel CdS NPs attached MOF material by using cadmium acetate as the CdS precursor and MIL-100(Fe) as the support [51]. The resulting CdS-MIL-100(Fe) nanocomposites showed remarkable photocatalytic efficiency in the selective oxidation of benzyl alcohol to benzaldehyde under visible light irradiation. The improved photocatalytic performance can be ascribed to the combined effect of enhanced light harvesting, high separation efficiency of photogenerated electron-hole pairs, as well as high dispersion of CdS NPs in MIL-100(Fe). The results indicate that the combination of semiconductors and MOF materials shows to be a promising approach for converting solar-energy into chemical energy. 

Polyoxometalates (POMs), a subclass of metal oxides, have attracted extensive attention in various fields because of their highly negative charges, various structural characteristics, and excellent redox ability [52]. Recently, POMs were encapsulated into MOFs. The specific interaction between the two led to reversible multiple electron transfer reactions without structural degradation of the framework. A porphyrinic MOF-545 containing the sandwich-type POM [(PW_9_O_34_)_2_Co_4_(H_2_O)_2_]^10^^−^ was recently used for visible-light-driven water oxidation [53]. The high photocatalytic activity of this hybrid material was speculatively ascribed to the synergistic effect of the photoactive porphyrin ligands and the cobalt POM’s catalytic sites that immobilized in the pore of MOF-545. Another example of POM-based MOF-101 hybrid material contains P_2_W_15_V_3_, P_2_W_17_Ni, or P_2_W_17_Co polyoxianions [54] which behaves as a photocatalyst in hydrogen production.

In some cases, MOF-based heterogeneous catalysts showed a reduced catalytic performance compared to homogeneous catalysts because the framework of MOFs can block the access of reactants to the catalytic sites. The reuse of MOF-based heterogeneous catalysts can extend the lifetime of the photocatalysts and reduce waste and contamination. The long term stability of MOFs as catalyst hosts under photocatalytic conditions is of great importance, and remains an issue. More efforts should be devoted to synthesizing robust MOFs that are stable in water or even acidic and basic solutions. 

### 2.3. MOFs as Precursors 

In recent years, MOFs have served as sacrificial templates or precursors in preparing more stable and conductive porous carbon, metal oxides, or porous carbon/metal oxides composite nanomaterials via a simple pyrolysis process [19]. MOFs have an inherent high porosity and a uniform dispersion of metal nodes in their network. Because of this, the MOF-derived nanomaterials keep the high porosity and the high surface area, and also show uniform heteroatom doping and adjustable morphology [55]. Therefore, the MOF-derived nanomaterials are promising candidates for catalytic applications. Zhao and coworkers successfully prepared TiO_x_/C composites by direct pyrolysis of MIL-125(Ti) under Ar atmosphere at different temperatures [56]. Among all the TiO_x_/C samples, T10, which was pyrolyzed at 1000 °C, possessed the highest photocatalytic activity for the photodegradation of methylene blue (MB). This was due to the reduced Ti_3_O_5_ composition, the conductive carbon support, as well as the high surface area. The incorporation of cocatalysts into TiO_2_ semiconductor photocatalysts has been applied for promoting charge separation and enhancing the photocatalytic performance. Xiong and coworkers synthesized a Cu/TiO_2_ octahedral-shell photocatalyst derived from Cu_3_(BTC)_2_/TiO_2_ core-shell structures (BTC = benzene-1,3,5-tricarboxylate) [57]. The Cu_3_(BTC)_2_ MOF not only serves as the sacrificial precursor to form the hollow structure but is also used as a Cu source to prepare the Cu/TiO_2_ composite. Because Cu can function as a cocatalyst, the Cu/TiO_2_ composites show improved electron-hole separation and can be used as a photocatalyst for hydrogen production

## 3. The Photocatalytic Applications of MOFs 

### 3.1. MOFs for Photocatalytic Degradation of Organic Pollutants

There are various approaches to remove organic pollutants from industrial wastewater: electrochemical oxidation, photocatalysis, adsorption, and biodegradation [58,59,60,61,62,63]. Photocatalytic degradation is considered as one of the most competitive methods for organic pollutants removal, due to its high efficiency, utilization of renewable solar energy, and environmental-friendliness [64]. In 2007, Garcia and coworkers demonstrated that MOF-5 exhibited photocatalytic activity for phenol degradation under UV light irradiation [65]. Since then, many MOF-based materials have been studied as photocatalysts for organic pollutants degradation (Table 1). For instance, MIL-53(M) (M = Fe, Al, Cr) was used to decolorize MB following first-order kinetics [66]. Recently, Wang and coworkers reported a pillared-layer MOF NNU-36 with broad-range visible light absorption and good chemical stability, which exhibits an efficient photocatalytic performance for aqueous Cr(VI) reduction and Rhodamine B (RhB) degradation [67]. Zhang et al. constructed two 3D MOFs [Cu(4,4’-bipy)Cl]*_n_* and [Co(4,4’-bipy)·(HCOO)_2_]*_n_* with photocatalytic activity for MB degradation under visible light irradiation [68]. Upon adding H_2_O_2_ electron acceptors, the photocatalytic performance of MB degradation was remarkably enhanced, following the LMCT mechanism. These results show that MOFs exhibit potential in photocatalytic organic pollutants degradation. However, it is still a great challenge to develop highly efficient MOF-based photocatalysts for organic pollutants degradation.

**Figure 3:**
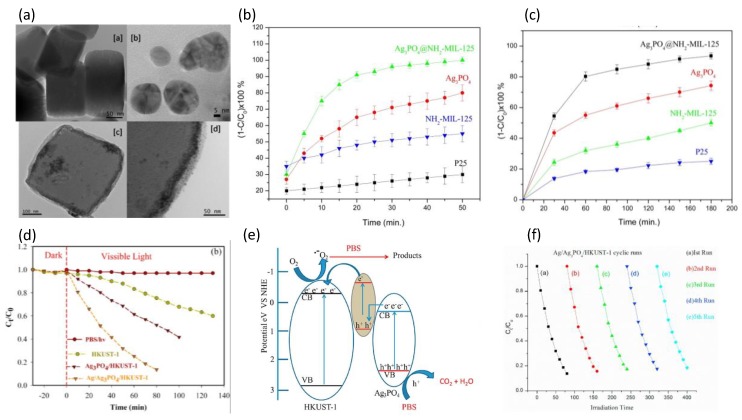
(**a**) TEM of (a) MIL-125-NH2, (b) Ag_3_PO_4_ NPs, (c) Ag_3_PO_4_@NH_2_-MIL-125, and (d) high magnification of the particle edge of Ag_3_PO_4_@NH_2_-MIL-125. (**b**) Photocatalytic decompositions of MB with Ag_3_PO_4_, NH_2_-MIL-125, Ag_3_PO_4_@NH_2_-MIL-125 composites and commercial TiO_2_ (P25) under visible-light irradiation. (**c**) Photocatalytic decompositions of RhB with Ag_3_PO_4_, NH_2_-MIL-125, Ag_3_PO_4_@NH_2_-MIL-125 composites and commercial TiO2 (P25) under visible-light irradiation. Reproduced with permission from Reference [69]. Copyright 2017 Elsevier B.V. (**d**) The degradation efficiency (C_t_/C_0_) of PBS in presence of Pristine HKUST-1, Ag_3_PO_4_/HKUST-1 and Ag/Ag_3_PO_4_/HKUST-1. (**e**) Schematic diagram showing the band structure and Z-Scheme separation of photoinduced electron hole pairs at the interface of the Ag/Ag_3_PO_4_/HKUST-1 catalyst under visible light irradiation. (**f**) The repeated experiments of photocatalytic degradation of PBS over the Ag/Ag_3_PO_4_/HKUST-1 catalyst. Reproduced with permission from Reference [70]. Copyright 2017 Elsevier B.V.

Several methods have been explored to improve the photocatalytic activity of MOF-based materials for organic pollutants degradation. For example, metal NPs loading and photocatalytically active composites modification. Karmaoui and coworkers modified the band gap of NH_2_-MIL-125(Ti) with Ag_3_PO_4_ NPs because of its narrow band gap [69]. The hybrid material NH_2_-MIL-125(Ti)@Ag_3_PO_4_ was synthesized by coating Ag_3_PO_4_ NPs on the edge of NH_2_-MIL-125(Ti) to form a core-shell structure, which was confirmed by the results of transmission electron microscopy (TEM) (Figure 3a). The band gap of NH_2_-MIL-125(Ti) in the hybrid material was decreased to 2.39 eV, indicating their potential for photocatalytic applications. The photocatalytic MB and RhB degradation under visible light irradiation were used to evaluate the photocatalytic activity of NH_2_-MIL-125(Ti)@Ag_3_PO_4_. As expected, NH_2_-MIL-125(Ti)@Ag_3_PO_4_ exhibits remarkably enhanced photocatalytic performance compared to P25 (P25 = Evonik commercial mixed anatase-rutile phase TiO2 nanophosphor), Ag_3_PO_4_ andNH_2_-MIL-125(Ti). This can be ascribed to the formation of an heterojunction between NH_2_-MIL-125(Ti) and Ag_3_PO_4_ (Figure 3b,c). Considering the surface plasmon resonance (SPR) of Ag NPs, Mehraj and coworkers developed a novel three-component photocatalyst Ag/Ag_3_PO_4_/HKUST-1 [70]. The deposition of Ag_3_PO_4_ NPs in this heterostructured system extends the light absorption to the visible region. Furthermore, the strong SPR effect of Ag NPs helps to boost the electron-hole separation at the interface of this composite, resulting in the drastically enhanced photocatalytic performance of HKUST-1. Photocatalytic degradation of Ponceau BS (PBS) was used to investigate the photocatalytic activity of the Ag/Ag_3_PO_4_/HKUST-1 system. It exhibited 87% degradation as compared to 60% by Ag_3_PO_4_/HKUST-1 and 40% by HKUST-1 (Figure 3d). The enhanced photocatalytic performance of the prepared system was attributed to the synergistic effects of the sequential energy transfer through the Z-scheme mechanism and the SPR effect of Ag NPs (Figure 3e). Additionally, the Ag/Ag_3_PO_4_/HKUST-1 system is highly stable and reusable (Figure 3f). These results indicate that the application of metal NPs on MOF-based materials is a potential approach to enhance the photocatalytic activity of MOFs.

Graphitic carbon nitride (g-C_3_N_4_) has been studied intensively because of its appealing electronic structure and high chemical stability [71]. More importantly, g-C_3_N_4_ possesses appropriate band positions and gap (2.7 eV) for light absorption up to 450 nm. Therefore, g-C_3_N_4_ can be used as a photocatalyst for organic pollutants degradation and many other reactions. Wen and coworkers designed a novel hybrid photocatalyst of protonated g-C_3_N_4_ coated MIL-100(Fe) frameworks through an in-situ protonation followed by a dip-coating procedure [72]. As compared with the parent materials, the protonated g-C_3_N_4_ coated MIL-100(Fe) material showed improved photocatalytic performance in MB and RhB degradation, as well as in oxidative denitrogenation for pyridine by molecular oxygen under visible light irradiation. The excellent photocatalytic activity of this hybrid material can be attributed to the enhanced absorption ability by introducing protonated g-C_3_N_4_ on MIL-100(Fe) frameworks and the enhanced photogenerated electron-hole separation through the coating effect. Another study on carbon nitrides and MOFs hybrid materials for photocatalysis was reported by Dontsva and coworkers [73]. In this study, the potassium poly(heptazine imide)/MIL-125-NH_2_ (PHIK/MIL-125-NH_2_) composites were prepared through the dispersion of both materials in water. The results of the surface ζ-potentials of the parent solids suggested that the driving forces of composite formation are the K^+^ ions diffusion from PHIK to MIL-125-NH_2_ and the electrostatic interactions between the solids. The formation of this composite was further confirmed by the analysis of FTIR, photoluminescence spectra, as well as SEM. The composites exhibited a remarkable enhanced photocatalytic performance in RhB degradation under blue light irradiation. The reaction rate of this composite was twofold higher than the reaction rate of the parent MOF compound and it displayed a sevenfold enhancement in comparison to the pristine PHIK. Based on the results of EPR studies and Mott-Schottky analysis, the excellent photocatalytic activity of the composite was due to the charge transfer from MIL-125-NH_2_ to PHIK. Except for carbon nitrides, many other kinds of photocatalytically active composites are being extensively explored to improve the photocatalytic activity of MOFs in recent years [74,75,76].

**Figure 4:**
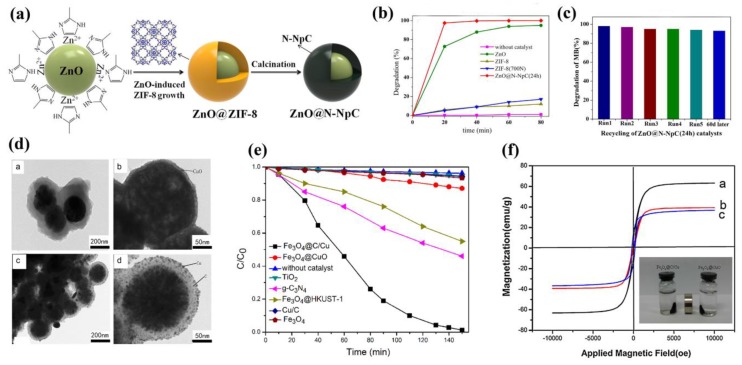
(**a**) Schematic illustration of ZnO@N-NpC formation. (**b**) Photodegradation cures of MB as a function of UV irradiation time in the presence of catalysts commercial ZnO, ZIF-8, ZIF-8(700N) and ZnO@N-NpC(24 h). (**c**) The MB photocatalysis repeatability test. Reproduced with permission from Reference [77]. Copyright 2017 Elsevier Inc. (**d**) TEM images of (a) Fe_3_O_4_@HKUST-1 core–shell microspheres, (b) Fe_3_O_4_@CuO, (c and d) Fe_3_O_4_@C/Cu. (**e**) Photodegradation of different catalytic conditions under visible light irradiation. (**f**) Hysteresis loops recorded at 300 K of (a) Fe_3_O_4_@CuO, (b) Fe_3_O_4_@C/Cu and (c) the as-prepared Fe_3_O_4_@HKUST-1 (inset: separation of Fe_3_O_4_@CuO and Fe_3_O_4_@C/Cu from solution under an external magnetic field). Reproduced with permission from Reference [78]. Copyright 2013 Elsevier B.V.

By calcination of MOFs, various carbons, metal or metal oxides, and nanomaterials with different properties can be easily fabricated. Chen and coworkers synthesized ZnO NPs with N-doped nanoporous carbon (N-NpC) via a simple approach of encapsulation and carbonization using ZIF-8 as the carbon source [77]. In the fabrication of ZnO@ZIF-8, ZnO NPs not only acts as the support, but also serves as the Zn source for synthesizing ZIF-8 (Figure 4a). The ZnO@N-NpC core-shell heterostructures were obtained after calcination under N_2_ atmosphere at 700 °C. As expected, the prepared ZnO@N-NpC core-shell composites exhibited excellent absorption and photocatalytic MB degradation over the pure ZnO. MB dyes were almost completely degraded in the presence of ZnO@N-NpC core-shell composites under UV light irradiation after 20 min (Figure 4b). Furthermore, this hybrid composite could be reused for five cycles (Figure 4c) and stored for 2 months, indicating its potential in practical photocatalytic applications. Xiao and coworkers successfully synthesized core-shell-structured Fe_3_O_4_@C/Cu and Fe_3_O_4_@CuO composites through direct calcinations of magnetic Fe_3_O_4_@HKUST-1 under N_2_ or air (Figure 4d) [78]. The analysis of UV-vis diffuse reflectance spectroscopy (UV-vis DRS) showed the calcined composites could absorb visible light up to 700 nm. The calculated band gap energy (*E*_g_) value of Fe_3_O_4_@C/Cu was around 1.75 eV, lower than that of Fe_3_O_4_@CuO (1.82 eV), g-C_3_N_4_ (2.7 eV) [79], and TiO_2_ (3.2 eV) [80]. This can be ascribed to the SPR effect of Cu NPs. Cu NPs can accept the photoinduced electrons from Fe_3_O_4_ microsphere, while the photoinduced holes remain on Fe_3_O_4_ microspheres, therefore promoting the effective charge separation and decreasing electron-hole recombination. As a result, the Fe_3_O_4_@C/Cu composites exhibited excellent photocatalytic activity for MB degradation in comparison with Fe_3_O_4_@CuO, g-C_3_N_4_, and TiO_2_ under visible light irradiation in the presence of H_2_O_2_ (Figure 4e). Furthermore, the magnetic Fe_3_O_4_@C/Cu composites could be easily separated from the reaction media with the help of an external magnetic field (Figure 4f) and be reused five times while preserving the reactivity under photocatalytic conditions. These results show that novel nanocomposites derived from MOF-based materials through a simple calcination procedure, show high stability and superior photocatalytic activity for organic pollutants degradation. This can be used for degrading organic pollutants from industrial waste water.

### 3.2. MOFs for Organic Photocatalysis

The use of MOF-based materials for light-induced organic transformations has attracted extensive interest due to the solar-energy based “green” organic synthesis condition. In comparison to other photocatalytic applications, photocatalytic transformations always need precise control of the adequate reaction rates and selectivity. Therefore, it is a great challenge to fabricate a MOF-based photocatalytic system with high selectivity. Due to the remarkable activity of TiO_2_ in photocatalysis [81], Ti-containing MOFs have been investigated for photocatalytic oxidation of amines, hydrazine, alkylphenols, alcohols and so on [82,83]. Mechanistic studies suggest that Ti^3+^ centers are generated upon UV-vis excitation, accompanying the oxidation of alcohols. When the highly active Ti^3+^ centers are oxidized into Ti^4+^, the O_2_ are reduced into superoxide diatomic ^·^O_2_^−^, which then reacts with the carbon-centered radicals to form aldehydes or imines. Zr-containing MOFs, particularly the UiO-type MOFs, are extensively explored in photocatalysis because of their ultra-high stability in water. In 2012, Wang and coworkers used NH_2_-UiO-66(Zr) as photocatalysts for aerobic oxygenation of various organic compounds, such as cyclic alkanes, olefins, and alcohols with high efficiency and selectivity [84]. The Fe-containing MOFs have received increasing attention in photocatalytic applications because the extensive Fe-O clusters in Fe-containing MOFs can be directly excited by visible light. Two Fe-containing MOFs, MIL-100(Fe) and MIL-68(Fe), were reported for photocatalytic hydroxylation of benzene to phenol with high selectivity under visible light irradiation [85]. A maximal benzene conversion of 30.6% was achieved under optimal conditions (H_2_O_2_:Benzene = 3:4, CH_3_CN:H_2_O = 1:1 (*v*/*v*)) over MIL-100(Fe) after 24 h irradiation. This work shows the potential of Fe-containing MOFs as photocatalysts for benzene hydroxylation with H_2_O_2_ as an oxidant, leading to a green and economical process for phenol production.

Incorporation of metalloligand complexes like Ru(bpy)_3_^2+^ and Ir(ppy)_2_(bpy)^+^ into MOFs can extend the MOF-based photocatalytic transformations to Aza-Henry reactions, oxidation of sulfides and arylboronic acids, as well as oxidative coupling of amines [86,87]. The resulting MOFs have exhibited slightly lower photocatalytic activity in comparison to the homogeneous catalysts, while excellent yields and reusability were achieved for these MOF materials.

**Figure 5:**
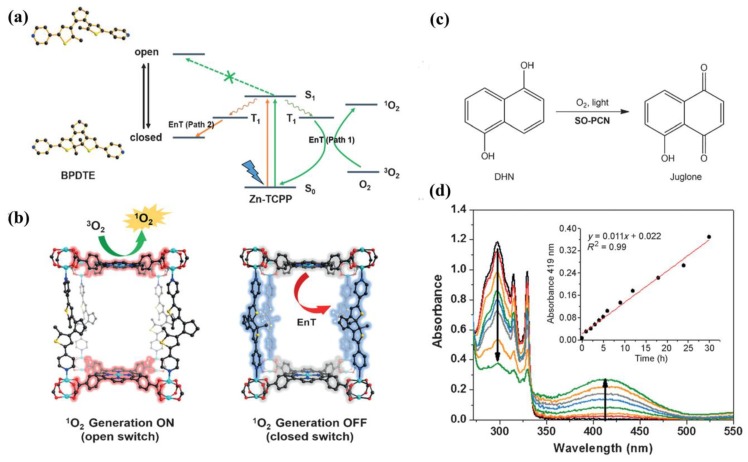
(**a**) Proposed mechanism of energy transfer (EnT) in SO-PCN. (**b**) Illustration of switching operation in SO-PCN. (**c**) Photo-oxidation of DHN catalyzed by SO-PCN in the presence of oxygen and light irradiation. (**d**) UV/Vis spectra of photo-oxidation of DHN in CH_3_CN catalyzed by SO-PCN. Inset: Absorbance of juglone (λ = 419 nm) as a function of reaction time. Reproduced with permission from Reference [89]. Copyright 2015 Wiley-VCH Verlag GmbH & Co. KGaA, Weinheim.

**Figure 6:**
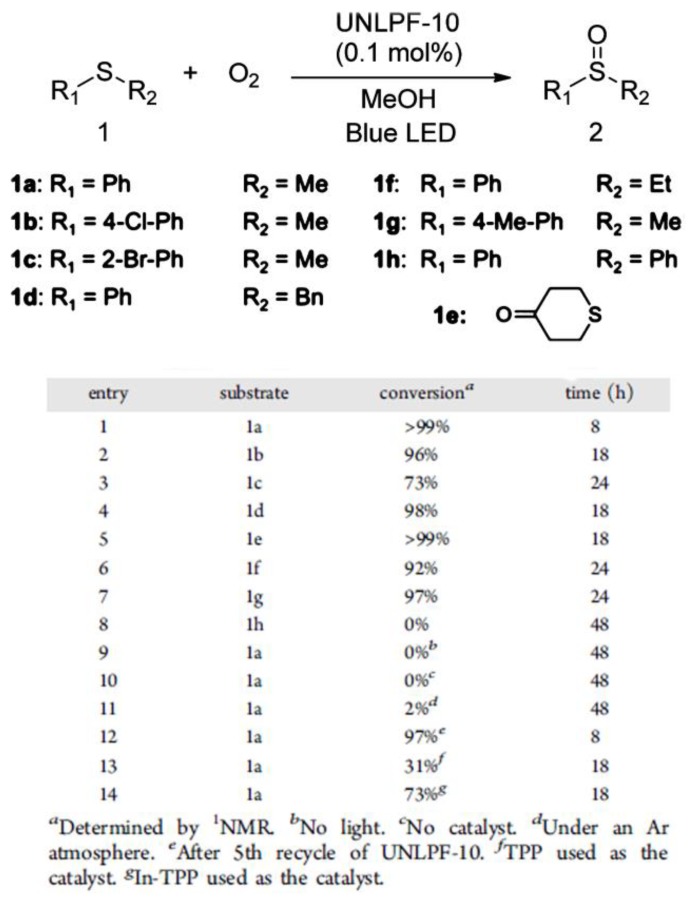
Photo-Oxygenation of Sulfides. Reproduced with permission from Reference [90]. Copyright 2014 American Chemical Society.

Porph-MOFs show great potential in photocatalysis. For instance, Wu and coworkers synthesized a tin-porphyin MOF [Zn_2_(H_2_O)_4_Sn^IV^(TPyP)(HCOO)_2_]·4NO_3_·DMF·4H_2_O (Sn^IV^TPyP = 5,10,15,20-tetra(4-pyridyl)-tin(IV)-porphyrin) [88] showing excellent photocatalytic activity for the oxygenation of sulfides and phenols with higher selectivity than that of the homogeneous catalyst Sn^IV^(OH)_2_TPyP. Zhou and coworkers designed a porph-MOF (SO-PCN) with 1,2-bis(2-methyl-5-(pyridin-4-yl)thiophen-3-yl)cyclopent-1-ene (BPDTE) as a photochromic switch and TCPP as a photosensitizer [89]. This exhibits reversible control of ^1^O_2_ generation and can be applied in 1,5-dihydroxynaphthalene (DHN) photo-oxidation (Figure 5). In 2014, Zhang and coworkers prepared an anionic porph-MOF UNLPF-10 with in-situ metalation in porphyrin using tetrakis 3,5-bis[(4-carboxy)-phenyl]phenylporphine (H_10_tbcppp) as organic linkers [90]. UNLPF-10 can be used as a photocatalyst for the selective oxygenation of sulfides with excellent yields (Figure 6). It also showed high stability and preserved its crystalline nature after reactions. These studies show that the immobilization of photoactive sites on/in MOFs can result in a remarkable photocatalytic performance for organic transformations.

**Figure 7:**
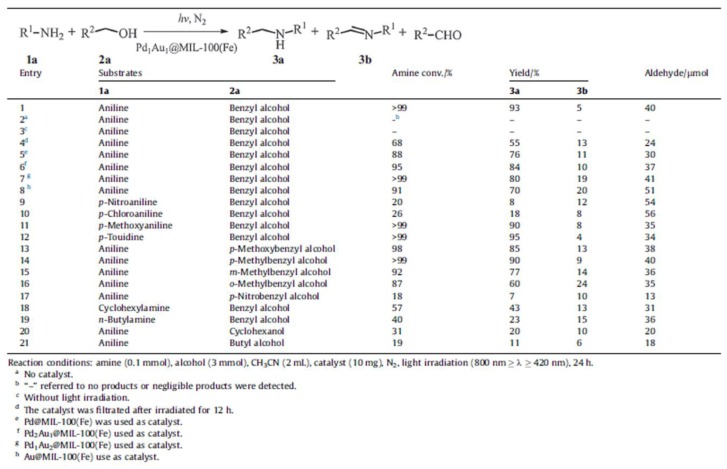
Light-induced catalytic performance for N-alkylation of amines with alcohols over Pd_1_Au_1_@MIL-100(Fe). Reproduced with permission from Reference [92]. Copyright 2018 Elsevier Inc.

In 2016, Li and coworkers prepared a Pd@MIL-100(Fe) catalytic system by a double-solvent impregnation, followed by a photo-reduction process [91]. TEM images revealed that Pd NPs are dispersed inside the MIL-100(Fe) cavity with an average size of 1.7 nm. After encapsulation of Pd NPs, the UV-vis DRS spectrum of Pd@MIL-100(Fe) exhibited an enhanced absorption in the range of 200–550 nm. This can be compared to the pure MIL-100(Fe), with the absorption edge extending to around 650 nm. Therefore, the Pd@MIL-100(Fe) composites show significant superior photocatalytic activity for N-alkyation of amines with alcohols under visible light irradiation. The Pd@MIL-100(Fe) catalytic system exhibited the highest conversion of aniline of 88%, and a selectivity to N-benzylaniline of 76% with the aniline/benzyl alcohol ratio of 1:30 after 24 h irradiation. Recently, the same group prepared a bimetallic PdAu@MIL-100(Fe) catalytic system for the light-induced tandem reaction between amines and alcohols to produce N-alkyl amines (Figure 7) [92]. This can be ascribed to the promoting effect in the photocatalytic alcohol-to-aldehyde dehydrogenation of metallic Au. Non-noble metal NPs incorporated MOF composites were also applied for photocatalytic transformations. Wu and coworkers reported that CdS-NH_2_-UiO-66 composites decorated CdS NRs on the surface of NH_2_-UiO-66 via a facile photo deposition approach [93]. This work showed that the CdS-NH_2_-UiO-66 composites can be used as a potential photocatalyst for the selective oxidation of alcohols to their corresponding aldehydes with O_2_ as the oxidant under visible light irradiation. The large specific surface area of NH_2_-UiO-66 and the effective charge separation could be responsible for the improved photocatalytic performance. 

### 3.3. MOFs for Photocatalytic CO_2_ Reduction

The solar transformation of CO_2_ into desirable organic products such as CO, methane (CH_4_), methanol (CH_3_OH), and HCOOH is a promising approach to reduce the green-house effect and produce renewable energy. Therefore, MOFs are very promising in the field of CCU (Carbon Capture and Utilization). Considerable research has been done in recent years (Table 2) [94].

Recently, Wang and coworkers reported a visible light-driven catalytic system using a cobalt-containing zeolitic imidazolate framework (Co-ZIF-9) as a robust MOF co-catalyst and [Ru(bpy)_3_]Cl_2_·6H_2_O as a photosensitizer [95]. This photocatalytic system could reduce CO_2_ to CO with triethanolamine (TEOA) as a sacrificial electron donor at 20 °C and 1 atm CO_2_. Upon visible-light irradiation, the CO and H_2_ production rates were 1.4 and 1.0 μmolmin^−1^, respectively. However, the CO_2_ reduction could not occur in the dark or without the ruthenium-based photosensitizer. Moreover, it was hindered drastically when the photocatalytic system was operated without Co-ZIF-9. Furthermore, the CO and H_2_ evolution decreased sharply when the residues of ZIF-9 after calcination at 1200 °C in helium gas were applied in this system. The results show that the framework of ZIF-9 plays a vital role in CO_2_ reduction through the promotion of the substrate concentration and carrier transfer. Later, the same group used nanoscale ZIF-67 instead of Co-ZIF-90 as the cocatalyst for CO_2_ splitting [96]. The new hybrid CO_2_ reduction system achieved an enhanced photocatalytic performance with a CO and H_2_ evolution rate of 37.4 and 13 μmol/30 min, respectively, which indicated that ZIF-67 was a novel and efficient cocatalyst for photocatalytic CO_2_ reduction. 

**Figure 8:**
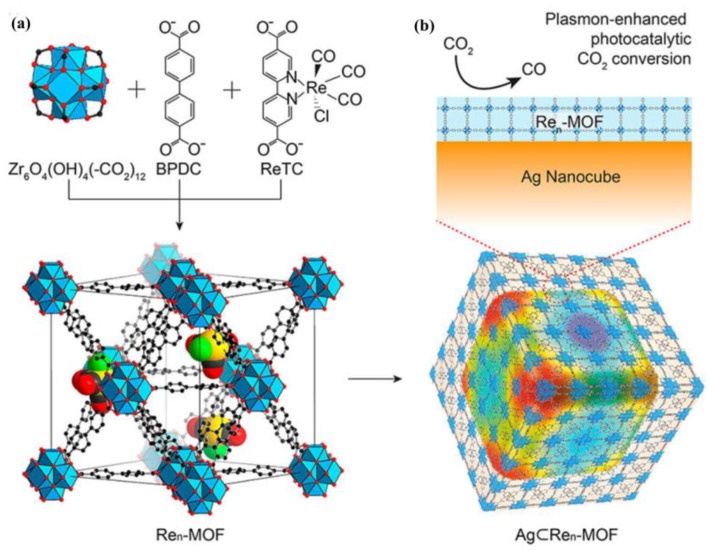
Structures of Re_n_-MOF and Ag⊂Re_n_-MOF for plasmon-enhanced photocatalytic CO_2_ conversion. (**a**) Zr_6_O_4_(OH)_4_(−CO_2_)_12_ secondary building units are combined with BPDC and ReTC linkers to form Re_n_-MOF. The structure of Re_3_-MOF identified from single-crystal X-ray diffraction is shown. The 12 coordinated Zr-based metal clusters are interconnected by 21 BPDC and three ReTC linkers in a face-centered cubic array. Atom labeling scheme: C, black; O, red; Zr, blue polyhedra; Re, yellow; Cl, green; H atoms are omitted for clarity. (**b**) Re_n_-MOF coated on an Ag nanocube for enhanced photocatalytic conversion of CO_2_. Reproduced with permission from Reference [98]. Copyright 2016 American Chemical Society.

Atomically dispersed photocatalysts, including mononuclear metal compounds or single metal atoms anchored on supports, exhibit the maximum efficiency of metal atoms and allow to investigate the photocatalytic process at the molecular level [97]. Yaghi and coworkers fabricated in 2017 a Re-containing UiO-67 (Re*_n_*-MOF) by covalently attaching Re^I^(CO)_3_(BPYDC)(Cl) (ReTC, BPYDC = 2,2′-bipyridine-5,5′-dicarboxylate) to a zirconium MOF for CO_2_-to-CO conversion (Figure 8a) [98]. The precise and quantitative control of the density of photoactive Re centers in the MOF unit could change the photocatalytic activity. Re_3_-MOF, in which each MOF cell unit contains three ReTCs, was found to exhibit the highest photocatalytic activity. Additionally, coating plasmonic Ag NPs on Re_3_-MOFs enhanced CO conversion by seven times under visible light irradiation with long-term stability up to 48 h (Figure 8b). This exceptional photocatalytic performance of CO_2_-to-CO conversion was ascribed to the synergistic effect of the spatially confined photoactive Re sites and the plasmonic Ag NPs. A Zr-MOF Zr_6_O_4_(OH)_4_(TCPP-H_2_)_3_ (MOF-525, TCPP = 4,4′,4′′,4′′′-(porphyrin-5,10,15,20-tetrayl) tetrabenzoate) constructed by Zr_6_ clusters and light-harvesting porphyrin linkers, was selected by Ye and coworkers as MOF support, because of its high CO_2_ capture capacity and visible-light utilization [99]. A new composite (MOF-525-Co) with single Co sites was generated by incorporating unsaturated Co centers into the porphyrin units. The active Co sites in MOF-525-Co were isolated by the porphyrin linkers and exposed to molecular CO_2_ simultaneously. As a result, MOF-525-Co showed significantly enhanced photocatalytic CO_2_ conversion. The CO and CH_4_ evolution rate of 200.6 and 36.76 mmolg^−1^h^−1^ respectively, were 3.13 times higher than those of the parent MOF-525 (CO: 64.02 mmolg^−1^h^−1^; CH_4_: 6.2 mmolg^−1^h^−1^). The incorporated single Co sites in MOF-525 enhanced the CO_2_ capture capacity and increased the charge separation efficiency in porphyrin linkers. This results in a significantly enhanced photocatalytic performance of CO_2_ conversion. These results indicate that the rational introduction of atomically dispersed photocatalysts into MOF frameworks is a promising approach for CO_2_ conversion.

**Figure 9:**
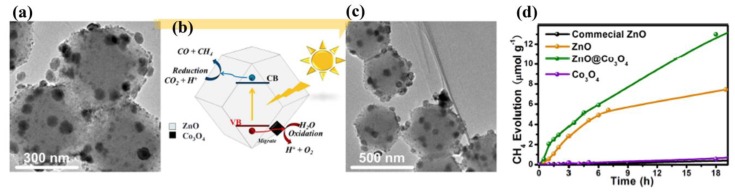
TEM images of ZnO@Co_3_O_4_ prepared from ZIF-8@ZIF-67: (**a**) before and (**c**) after photocatalytic CO_2_ reduction. Schematic illustration of the photocatalytic CO_2_ reduction with (**b**) ZnO@Co_3_O_4_. (**d**) CH_4_ evolution over various samples under UV-vis irradiation. Reproduced with permission from Reference [100]. Copyright the Royal Society of Chemistry 2016.

Besides their use as cocatalysts or supports, MOF-derived nanomaterials are also very promising for photocatalytic CO_2_ conversion. Wang et al. converted the core-shell ZIF-8@ZIF-67 crystals into a novel porous ZnO@Co_3_O_4_ composite through a seed-mediated growth process followed by a two-step calcination process (Figure 9a–c) [100]. The resultant porous ZnO@Co_3_O_4_ composite exhibited a much higher photocatalytic performance of CO_2_ conversion with a CH_4_ evolution rate of 0.99 μmolg^−1^h^−1^. This was a 66fold enhancement compared to the commercial ZnO (0.015 μmolg^−1^h^−1^) and 367fold enhancement compared to commercial TiO_2_ (P25) (0.0027 μmolg^−1^h^−1^) (Figure 9d). The exceptional photocatalytic activity of ZnO@Co_3_O_4_ composite was ascribed to its advantageous porous structure and the cocatalytic function of Co_3_O_4_ NPs. Additionally, Co_3_O_4_ NPs can significantly decrease the ZnO photocorrosion and thus, improve its photocatalytic stability. Zhang and coworkers prepared a ZnO/NiO porous hollow sphere with sheet-like subunits through thermal treatment of Ni-Zn MOFs for CO_2_ conversion [101]. The NiO content in the ZnO/NiO porous hollow spheres was optimized to improve the special surface, CO_2_ uptake, and the electron-charge separation of the composites. The excess NiO decreased the incident light absorption and accelerated charge recombination, therefore decreasing the photocatalytic activity. As a result, ZnO/NiO composites with 30% Ni^2+^, denoted as ZN-30, exhibited excellent photocatalytic CO_2_ conversion with the CH_3_OH evolution of 1.57 μmolg^−1^h^−1^. This was due to the highly specific surface area, CO_2_ capture capacity, and increased light absorption of the porous hollow structure. Additionally, the authors believed that the n-type ZnO and n-type NiO were derived from Zn-Ni MOFs mixed together homogeneously, leading to the formation of various p-n heterojunctions which could boost the electron-hole separation. These results demonstrate the design of a novel heterogeneous composite with a special structure, by using MOFs as templates, providing new insights to fabricate new photocatalysts with high CO_2_ reduction performance.

### 3.4. MOFs for Water Oxidation

Photocatalytic water splitting into hydrogen and oxygen is a promising and effective strategy to transfer solar energy into chemical energy. To date, many MOF-based materials have been employed as photocatalysts for hydrogen production (Table 3). Du and coworkers reported a Cu_2_I_2_-based MOF, Cu-I-bipy, for hydrogen production by UV light with TEOA as a sacrificial agent [102]. It exhibited highly efficient photocatalytic hydrogen evolution with an average rate of 7.09 mmolg^−1^h^−1^. This exceeds most of the reported MOF-based materials [103]. The Cu_2_I_2_ clusters of Cu-I-bipy, serving as the photoelectron generators, can accelerate the Cu(I) hydride interaction, thus providing redox reaction sites for hydrogen production, which is responsible for the excellent photocatalytic activity for hydrogen generation. Furthermore, Cu-I-bipy can be reused at least five times with negligible loss of catalytic activity, indicating it could be a practical application in water splitting. 

Integration of appropriate co-catalysts like metal NPs, POMs, metal oxides and carbon nitrides has proved to be a potential approach to improve the photocatalytic performance for water splitting. Wang and coworkers reported a NH_2_-MIL-125(Ti)/0.75CN/Ni_15.8_Pd_2.1_ photocatalytic system, exhibiting enhanced photocatalytic activity for hydrogen production under visible light irradiation [104]. It exhibited a high hydrogen evolution rate of 8.7 mmolg^−1^h^−1^, 332 and 1.3 times higher than those of NH_2_-MIL-125(Ti)/0.75CN and NH_2_-MIL-125(Ti)/Ni_15.8_Pd_4.1_, respectively. The improved photocatalytic activity of hydrogen production was ascribed not only to the strong light-absorbing capacity and increased charge transfer of loaded NiPd NPs, but also to the enhanced electron holes separation of heterostructure between NH_2_-MIL-125(Ti) and CN.

**Figure 10:**
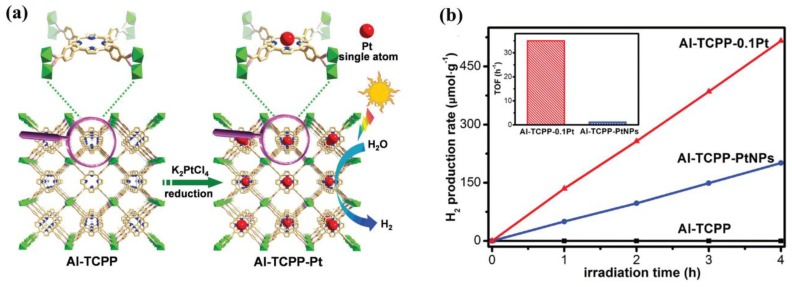
(**a**) Schematic illustration showing the synthesis of Al-TCPP-Pt for photocatalytic hydrogen production. (**b**) Photocatalytic hydrogen production rates of Al-TCPP, Al-TCPP-PtNPs, and Al-TCPP-0.1Pt (inset: the calculated TOFs of Al-TCPP-PtNPs and Al-TCPP-0.1Pt). Reproduced with permission from Reference [105]. Copyright 2018 WILEY-VCH Verlag GmbH & Co. KGaA, Weinheim.

Due to the maximized atomic efficiency, single-atom catalysts have exhibited excellent catalytic activity for various reactions, such as electrocatalysis, oxidation, water-gas shift, and hydrogenation. A highly stable porph-MOF Al-TCPP, formulated as (AlOH)_2_H_2_TPCC, was used as the support to anchor Pt(II) into the porphyrin centers (Figure 10a) [105]. Thus, a single Pt atom catalyst (Al-TCPP-Pt) was easily synthesized through a simple reduction process of Al-TCPP-Pt(II). As expected, Al-TCPP-0.1Pt exhibited an excellent photocatalytic performance for hydrogen evolution under visible irradiation, using TEOA as the sacrificial agent. The hydrogen evolution rate of Al-TCPP-0.1Pt was 129 μmolg^−1^h^−1^ and the calculated turnover frequency (TOF) of Al-TCPP-0.1Pt reached 35 h^−1^, 30 times higher than that of Al-TCPP-PtNPs (Figure 10b). The results of spectroscopic characterizations and DFT calculations confirmed that the single Pt atoms anchored into porphyrin centers of Al-TCPP open a channel for highly efficient electron transfer, and enhance the hydrogen binding energy, thus resulting in the enhanced photocatalytic performance of hydrogen evolution.

Recently, Lin and coworkers incorporated a Ni-containing POM [Ni_4_(H_2_O)_2_(PW_9_O_34_)_2_]^10−^ (Ni_4_P_2_) into highly stable [Ir(ppy)_2_(bpy)]^+^-derived UiO-MOFs (MOF-1) or [Ru(bpy)_3_]^2+^-derived UiO-MOFs (MOF-2) [106]. Ni_4_P_2_-MOF-1 exhibited excellent catalytic activity of hydrogen evolution in an acidic aqueous solution (pH = 1.2) with MeOH as the sacrificial electron donor under visible light irradiation. The hydrogen evolution rate of Ni_4_P_2_-MOF-1 was 4.4 mmolg^−1^h^−1^, and the turnover number (TON) of Ni_4_P_2_-MOF-1 reached 1476 in 72 h irradiation. However, Ni_4_P_2_-MOF-2 only produced trace amounts of hydrogen after 20 h irradiation under identical conditions. They believed that the proximity of Ni_4_P_2_ to multiple photosensitizers in Ni_4_P_2_-MOF realizes the multi-electron transfer and enhances the photocatalytic hydrogen evolution performance. Electrochemical and photophysical studies revealed that Ni_4_P_2_ can only oxidatively quench the excited state of [Ir(ppy)_2_(bpy)]^+^ as the initiating step of hydrogen evolution, resulting in the drastic differences of photocatalytic performance between the two Ni_4_P_2_-MOF systems.

**Figure 11:**
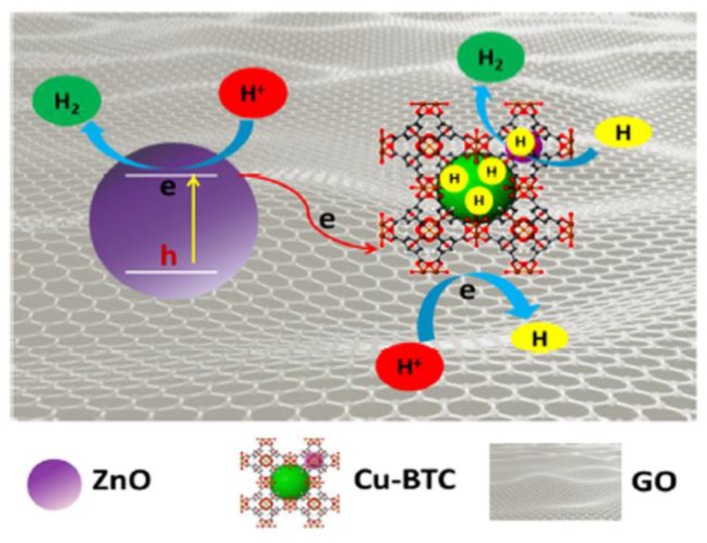
Schematic illustration of the electrostatic interaction assembly of ZnO/GO and Cu-BTC and its photocatalytic H_2_ evolution mechanism. Reproduced with permission from Reference [74]. Copyright Tsinghua University Press and Springer-Verlag GmbH Germany 2017.

To use semiconductor NPs as cocatalysts for photocatalytic hydrogen evolution, Ao and coworkers reported a series of heterostructured ZnIn_2_S_4_@NH_2_-MIL-125(Ti) composites with ZnIn_2_S_4_ nanosheets highly dispersed on the surface of NH_2_-MIL-125(Ti) [107]. The heterostructure between ZnIn_2_S_4_ nanosheets and NH_2_-MIL-125(Ti) could have increased the capacity of electron transfer and promoted the photogenerated charge separation, resulting in the enhanced photocatalytic performance of hydrogen evolution. Tang and coworkers encapsulated the Cu-BTC MOF into a ZnO/graphene oxide (GO) photocatalytic system through electrostatic interaction to form the electrostatic interaction assembly of ZnO/GO and Cu-BTC, which exhibited enhanced photocatalytic activity of hydrogen evolution [74]. In this prepared heterostructure, ZnO acted as the photoelectron generator, and GO served as the channel of photoelectron transfer from ZnO to Cu-BTC as well as the supporting matrix for ZnO and Cu-BTC (Figure 11). The electron spin resonance (ESR) results have revealed that the Cu-BTC could extend the lifetime of free radicals and boost the H recombination to form H_2_, enhancing the photocatalytic performance of hydrogen evolution. As a result, the electrostatic interaction assembly of ZnO/GO and Cu-BTC showed the highest hydrogen evolution rate of 129 μmolg^−1^h^−1^, which is ninefold and threefold higher than that of ZnO/GO and ZnO/(Cu-BTC)/GO complex, respectively. 

## 4. Conclusions

In this review, we have discussed the functions of MOF materials in the photoredox chemical systems. MOFs can be used as photocatalysts due to the facile modification of organic linkers with photocatalytic active groups such as amine and porphyrin. In addition, the high porosity of MOFs makes them capable to act as hosts for photoredox species, like precious metals, semiconductor NPs, and POMs, providing additional possibilities for photocatalytic applications. In recent years, MOFs have served as sacrificial templates or precursors in preparing more stable and conductive porous carbon, metal oxides, or porous carbon/metal oxides composite nanomaterials via simple pyrolysis processes. MOFs have an inherent high porosity and a uniform dispersion of metal nodes in their network. Because of this, the MOF-derived nanomaterials keep their high porosity and the high surface area, and show uniform heteroatom doping and adjustable morphology. Therefore, the MOF-derived nanomaterials are promising candidates for photocatalytic applications. The typical applications of MOFs in heterogeneous photocatalysis were summarized in detail, indicating that MOFs are promising candidates for heterogeneous photocatalysis. These applications were including pollutants degradation, organic transformations, CO_2_ reduction, and water splitting. There are still some problems that need to be solved. Only a tiny fraction of the many MOFs that have been reported are suitable for photocatalysis. Therefore, new MOFs with redox active metals and/or functional organic ligands should be designed and fabricated for heterogeneous photocatalysis. The photocatalytic efficiencies of MOFs do not yet meet the requirements for practical applications. It is of great importance to improve the photocatalytic efficiencies of MOFs. Finally, cost-effective photocatalytic systems should avoid the use of expensive precious metals, and replace them by abundant transition metals or by metal-free variants.

## Figures and Tables

**Table 1:** A summary of MOFs mentioned in this review for pollutant degradation.

MOF	Cocatalyst	Light Source	Electron Acceptor	Pollutant Degradation	Ref.
MOF-5	/	UV light	/	Phenol degradation	[59]
MIL-53	/	UV-vis light	H_2_O_2_, KBrO_3_, (NH_4_)_2_S_2_O_8_	MB degradation	[60]
NNU-36	/	Visible light	H_2_O_2_	Cr(VI) reduction RhB degradation	[61]
[Cu(4,4’-bipy)Cl]*_n_* [Co(4,4’-bipy)·(HCOO)_2_]*_n_*	/	Visible light	H_2_O_2_	MB degradation	[62]
NH_2_-MIL-125(Ti)	Ag_3_PO_4_	Visible light	/	MB and RhB degradation	[63]
HKUST-1	Ag, Ag_3_PO_4_	Visible light	/	PBS degradation^.^	[64]
MIL-100(Fe)	g-C_3_N_4_	Visible light	/	MB and RhB degradation	[66]
MIL-125-NH_2_	potassium poly(heptazine imide)	Visible light	/	denitrogenation for pyridine	[67]
MIL-125-NH_2_	CTAB	Visible light	Visible light	RhB degradation	[69]

**Table 2:** A summary of MOFs mentioned in this review for CO_2_ reduction.

MOF	Cocatalyst	Photosensitizer	Light Source	Sacrificial Agent	CO_2_ Reduction	Ref.
Co-ZIF-9	/	[Ru(bpy)_3_]Cl_2_·6H_2_O	Visible light	Triethanolamine	CO 1.4 μmol min^−1^ H_2_ 1.0 μmol min^−1^	[89]
ZIF-67	/	[Ru(bpy)_3_]Cl_2_·6H_2_O	Visible light	Triethanolamine	CO 1.25 μmol min^−1^ H_2_ 0.43 μmol min^−1^	[90]
Re_n_-MOF	Ag NPs	/	Visible light	Triethanolamine	CO	[92]
Zr_6_O_4_(OH)_4_(TCPP-H_2_)_3_	Single Co sites	/	Visible light	Triethanolamine	CO 200.6 mmolg^−1^ h^−1^ CH_4_ 36.76 mmolg^−1^ h^−1^	[93]
ZIF-8@ZIF-67	/	/	UV-vis light	/	CH_4_ 0.99 μmolg^−1^ h^−1^	[94]
Ni-Zn MOFs	/	/	Full-spectrum	/	CH_3_OH 1.57 μmolg^−1^ h^−1.^	[95]

**Table 3:** A summary of MOFs mentioned in this review for water oxidation.

MOF	Cocatalyst	Light Source	Sacrificial Agent	H2 Evolution (mmol·g^−1^·h^−1^)	Ref.
(Cu_3_(BTC)_2_(H_2_O)_3_)	ZnO/GO	UV light	Methanol	0.129	[68]
Cu-I-bipy	/	UV light	Triethanolamine	7.09	[96]
NH_2_-MIL-125(Ti)	0.75CN/Ni_15.8_Pd_4.1_	Visible light	Triethanolamine	7.84	[98]
(AlOH)_2_H_2_TCPP	single Pt atoms (0.07 wt%)	Visible light	Triethanolamine	0.129	[99]
[Ir(ppy)_2_(bpy)]^+^-derived UiO-MOF	[Ni_4_(H_2_O)_2_(PW_9_O_34_)_2_]^10−^	Visible light	Methanol	4.4	[100]
NH_2_-MIL-125(Ti)	ZnIn_2_S_4_	Visible light	Na_2_SO_3_, Na_2_S	2.2	[101]

## Abbreviations

BDC-NH_2_	2-amino-benzene-1,4-dicarboxylate
Bipy	4,4′-bipyridine
BPDTE	1,2-bis(2-methyl-5-(pyridin-4-yl)thiophen-3-yl)cyclopent-1-ene
bpy	2′2-bipyridine
BTC	benzene-1,3,5-tricarboxylate
CB	conduction band
CH_4_	methane
CH_3_OH	methanol
CO_2_	carbon dioxide
DHN	1,5-dihydroxynaphthalene
*E*_g_	band gap energy
ESR	electron spin resonance
g-C_3_N_4_	graphitic carbon nitride
H_4_L1	bis(3,5-dicarboxyphenyl)isophthalamide
H_2_O	water
H_10_tbcppp	3,5-bis[(4-carboxy)-phenyl]phenylporphine
H_2_TCPP	*meso*-tetra(4-carboxyl-phenyl) prophyrin
LMCT	ligand-to-metal charge transfer
MB	methylene blue
MOFs	metal organic frameworks
*N*-NpC	*N*-doped nanoporous carbon
NPs	nanoparticles
NRs	nanorods
P25	Degussa commercial mixed anatase-rutile phase TiO2 nanophosphor
PBS	Ponceau BS
PHIK	potassium poly(heptazine imide)
POMs	polyoxometalates
ppy	2-phenylpyridine
RhB	Rhodamine B
Sn^IV^TPyP	5,10,15,20-tetra(4-pyridyl)-tin(IV)-porphyrin)
SPR	surface plasmon resonance
TCPP	4,4′,4′′,4′′′-(porphyrin-5,10,15,20-tetrayl) tetrabenzoate
TEM	transmission electron microscopy
TEOA	triethanolamine
TOF	turnover frequency
TON	turnover number
UV-vis DRS	UV-vis diffuse reflectance spectroscopy
ZIF	Zeolitic imidazolate framework
